# Liposomal Nanosystems in Rheumatoid Arthritis

**DOI:** 10.3390/pharmaceutics13040454

**Published:** 2021-03-27

**Authors:** Margarida Ferreira-Silva, Catarina Faria-Silva, Pedro Viana Baptista, Eduarda Fernandes, Alexandra Ramos Fernandes, Maria Luísa Corvo

**Affiliations:** 1 Instituto de Investigação do Medicamento (iMed.ULisboa), Faculdade de Farmácia, Universidade de Lisboa, Av. Prof. Gama Pinto, 1649-003 Lisbon, Portugal; ana.m.silva@campus.ul.pt (M.F.-S.); ana.catarina.silva@campus.ul.pt (C.F.-S.); 2 Unidade de Ciências Biomoleculares Aplicadas UCIBIO, Departamento Ciências da Vida, Faculdade de Ciências e Tecnologia, Universidade Nova de Lisboa, Campus de Caparica, 2829-516 Caparica, Portugal; pmvb@fct.unl.pt; 3 Associated Laboratory for Green Chemistry of the Network of Chemistry and Technology (LAQV, REQUIMTE), Laboratory of Applied Chemistry, Department of Chemical Sciences, Faculty of Pharmacy, University of Porto, Rua de Jorge Viterbo Ferreira, 228, 4050-313 Porto, Portugal; egracas@ff.up.pt

**Keywords:** rheumatoid arthritis, drug delivery nanosystems, liposomes, passive targeting, active targeting

## Abstract

Rheumatoid arthritis (RA) is an autoimmune disease that affects the joints and results in reduced patient quality of life due to its chronic nature and several comorbidities. RA is also associated with a high socioeconomic burden. Currently, several available therapies minimize symptoms and prevent disease progression. However, more effective treatments are needed due to current therapies’ severe side-effects, especially under long-term use. Drug delivery systems have demonstrated their clinical importance—with several nanocarriers present in the market—due to their capacity to improve therapeutic drug index, for instance, by enabling passive or active targeting. The first to achieve market authorization were liposomes that still represent a considerable part of approved delivery systems. In this manuscript, we review the role of liposomes in RA treatment, address preclinical studies and clinical trials, and discuss factors that could hamper a successful clinical translation. We also suggest some alterations that could potentially improve their progression to the market.

## 1. Rheumatoid Arthritis

Rheumatoid arthritis (RA) is an immune-mediated chronic inflammatory disease characterized by chronic inflammation of the joint synovium and progressive joint destruction often associated with persistent arthritic pain, swelling and stiffness [1,2]. This disorder affects 1% of the adult population in Europe and the USA, with an incidence approximately 75% higher in women than in men [3,4]. The precise cause of RA remains uncertain, but it is has been generally considered that the crucial factor is an immunological response against the tissue that lines the joints [1,5,6]. Its chronic progression results in joint inflammation that can progress to joint destruction. Extra-articular manifestations, such as rheumatoid nodules and pulmonary vasculitis, can also occur, causing a decline in the quality and life expectancy of patients and increasing the comorbidity risk (e.g., metabolic and psychological disorders) [4,7,8]. Besides individual consequences, there is also a concomitant socioeconomic burden associated with the medical costs and the reduced work capability [2,9].

Due to the severe progression of RA, a fast diagnosis is crucial to initiate treatment before irreversible joint damage might happen [3,10,11]. Nevertheless, a fast differential diagnosis of RA is difficult to accomplish since symptoms are common to other types of arthritis or rarer autoimmune conditions, such as connective tissue diseases [9].

### Pathophysiology

**Figure 1:**
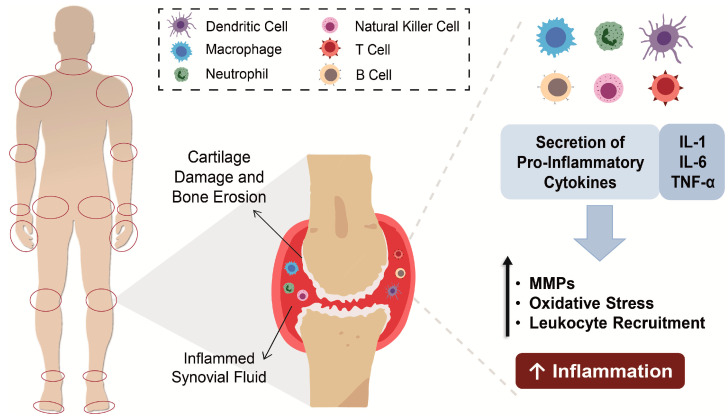
Overview of rheumatoid arthritis (RA) pathogenesis: inflammatory cells produce proinflammatory markers (e.g., cytokines) in the inflamed synovium of joints, enhancing the production of metalloproteinases (MMPs) that cause bone erosion; increasing the oxidative stress by production of reactive oxygen or nitrogen species (ROS, RNS respectively) and recruiting more leukocytes into the joint, exacerbating the inflammation. IL—interleukin; TNF-α—tumor necrosis factor-alpha.

The synovial fluid, produced by the synovium, acts as a lubricant in body joints and supplies cartilage with nutrients and metabolites [12]. In RA, the inflamed synovium is filled with inflammatory cells both from the innate immune system (e.g., monocytes, neutrophils, dendritic cells, macrophages, fibroblasts and innate lymphoid cells) and adaptive immune system (e.g., T-helper cells, B cells, and plasma cells) [9,13]. Upon activation, inflammatory cells release proinflammatory cytokines (e.g., tumor necrosis factor α, interleukin-1 and -6) and secrete matrix metalloproteinases and prostaglandins into the synovial fluid [12]. In sequence, cytokines act as recruiting agents, activating endothelial cells and enhancing the accumulation of inflammatory cells, with consequent exacerbation of inflammation in synovial tissues, while secreted matrix metalloproteinases and prostaglandins cause the degradation of cartilage and bones [13,14] (Figure 1). The progression of this disease from one arthritic joint to an unaffected joint has been attributed to activated fibroblasts [15].

## 2. Therapeutic Strategies Used in the Clinic

Since inflammation is the driving force in RA development, its suppression or attenuation is the main therapeutic strategy to improve symptoms, preserve the structural integrity of the joints and maintain patient quality of life [7]. To date, RA does not have a cure, and the available drugs are used to attenuate the symptoms and maintain patients with a functional life [13,16]. To achieve remission, it is crucial to initiate therapy within three months of disease onset [17]. When RA is in an advanced stage, usually the goal is not remission but to minimize disease activity/burden [18]. RA drugs are mainly divided into three classes: (i) nonsteroidal anti-inflammatory drugs (NSAIDs) that are usually prescribed for management of pain, stiffness and inflammation, improving patient overall physical function [3,19]; (ii) corticosteroids, also with anti-inflammatory, anti-angiogenic and immunoregulatory properties, allowing to promote the decrease of expression of cellular adhesion molecules and cytokines on endothelial cells and thus preventing joint erosions [20], and (iii) disease-modifying antirheumatic drugs (DMARDs), used to prevent joint damage [21]. Personalized treatment decisions should be based on the number of disease events, and regular follow-up visits may be needed, especially when RA is active [6]. Nonetheless, efficient predictors of patient response to the different drugs, according to the disease stage, are still needed [7]. Indeed, NSAIDs are not able to alter the progression of RA when used alone since they do not interfere with joint damage, and their long-term use is associated with gastrointestinal, cardiovascular and renal risks [22,23]. Corticosteroids are usually used in the early stages of RA, as temporary adjunctive therapy until DMARDs exert their effects, or as chronic adjunctive therapy when control of RA is not achieved with the other types of drugs [6,18]. Nonetheless, corticosteroids are associated with serious long-term side effects, such as osteoporosis, hypertension, diabetes, obesity, avascular necrosis, growth retardation, cataracts and muscle wasting [24]. When administered intravenously, they have a rapid clearance and a large distribution volume, with a higher dosage being necessary for an effective drug concentration at the inflamed sites [25]. While NSAIDs only control the symptoms, DMARDs decrease the structural damage progression in joints, being indicated when erosions or narrowing of joints space in X-rays are visible [21,26]. Currently, DMARDs are the standard drugs prescribed to newly diagnosed patients, along with NSAIDs or low-dose corticosteroids that decrease swelling and pain, since DMARDs usually take several weeks or months to demonstrate a clinical effect [27]. Until the 1980s, the standard DMARDs were gold salts (intramuscular), which are no longer used, due to their side effects, limited efficacy and slow action [28].

Nowadays, DMARDs can be divided into two main classes: synthetic or biological molecules, and these classes can be further subdivided into conventional or targeted synthetic DMARDs and in the biological originator and biosimilar DMARD [21]. Conventional synthetic DMARDs include sulfasalazine; penicillamine; antimalarials (hydroxychloroquine); gold compounds (auranofin), and immunosuppressors (methotrexate (MTX); leflunomide; azathioprine; cyclosporin A; cyclophosphamide), while targeted synthetic DMARDs comprise Janus kinase (JAK) inhibitors (tofacitinib, baricitinib, upadacitinib and filgotinib) [16,29]. From all the therapeutic options listed above, MTX is considered the first-line drug for most RA patients due to its high efficacy and the possibility to control its side effects with the prophylactic use of folates [26,30].

Biological originator DMARDs used in RA treatment can be subdivided into four classes. The tumor necrosis factor (TNF) inhibitors (etanercept, infliximab, adalimumab, certolizumab pegol and golimumab) that decrease the inflammatory response; T-cell costimulatory blocker (abatacept) that interferes with the interactions between antigen-presenting cells and T cells; the B cell depleting agent (rituximab) that leads to a rapid and sustained depletion of circulating B cells, reducing RA progression and the interleukin receptor inhibitors for interleukin-6 (IL-6) (tocilizumab and sarilumab) and interleukin-1 (IL-1) (anakinra) that decrease inflammation and RA progression [6,31]. The other group of biological DMARDs includes biosimilars of adalimumab, etanercept, infliximab and rituximab [16]. All the drugs listed in these groups are currently approved by FDA and EMA, according to their official websites (https://www.fda.gov/ and https://www.ema.europa.eu/, accessed on 15 February 2021).

Synthetic DMARDs have been associated with some undesirable side effects, namely in the gastrointestinal system (e.g., vomiting and diarrhea), in the central nervous system (e.g., headaches, dizziness and insomnia) or damages in skin and hair, while biological DMARDs can enhance the risk of infections, malignancy, anaphylaxis or autoimmune syndromes [27]. Biosimilars of some of the previously mentioned biological DMARDs are in different stages of development or already in the market [27]. For a more detailed description of the therapeutic recommendations followed by the American College of Rheumatology, the European League Against Rheumatism, and the Asia-Pacific League of Associations for Rheumatology consult [16,18,32].

Despite the wide variety of drugs for RA, their benefits are only temporary due to the off-target toxicity associated with long-term use [1]. This drawback is especially important when there is a systemic administration because of unfavorable pharmacokinetic properties (rapid clearance rate and unspecific distribution profile) that lead to more frequent administrations of high doses [33]. When only a couple of joints are affected by RA, or when they do not present a satisfactory response to the systemic administration, clinicians can use intra-articular injections as an alternative route, increasing local drug concentration in joints, minimizing the necessary doses and off-target side effects [34,35]. Additionally, these injections improve the delivery of therapeutic agents with low oral bioavailability, such as proteins and genetic material [34]. However, the intraarticular route has the drawback of rapid clearance of the injected agent, which leads to a higher frequency of joint needling, resulting in infection, joint disability, post-injection flare and intolerance of the patients [27].

### Tapering Therapy in Remission

With all the therapeutic strategies previously listed, RA remission is a more attainable goal—mainly when RA is in an early-stage, and the therapy starts soon after the onset of the disease [17]. The management of these patients is crucial to assure that no regression into an active RA is observed [36]. Currently, treatment guidelines suggest that clinicians should consider tapering therapy [16,18].

## 3. Biomarkers for Active Targeting in Rheumatoid Arthritis

A wide variety of targets have been explored for RA therapy associated with the vast number of factors involved in the inflammation of the synovial fluid. One of them is the folate receptor (FR-β) that is overexpressed in synovial macrophages of the inflamed joints. The ease chemical conjugation of folate to other molecules through the γ-carboxyl group allows the development of folate conjugates that are internalized through receptor-mediated endocytosis [37]. Other studies have identified the CD44 receptor (also overexpressed in the synovial lymphocytes, macrophages and fibroblasts of RA patients) as a possible target via conjugation with its ligand: hyaluronic acid [38].

Angiogenesis is of extreme importance in chronic inflammatory diseases: the newly formed blood vessels, a consequence of local hypoxia and growth factor production at inflamed joints [39], allow the permeation of the inflammatory cells into the inflamed tissue [40]. Furthermore, it is known that angiogenic factors stimulate the expression of adhesion molecules and inflammatory cytokines and chemokines in endothelia [41]. Within the angiogenic factors, αvβ3 integrins and vascular endothelial growth factor (VEGF) have been studied as therapeutic targets in RA. αvβ3 integrins are cell surface receptors expressed in the newly formed blood vessels in the RA synovium [39]. These integrins are essential in synovial angiogenesis, making them a potential target for RA treatment through their blockage [42]. Besides integrins, VEGF and its receptors also stimulate vascular permeability and angiogenesis and are overexpressed in inflammation [41,43,44]. Due to their crucial role, VEGF and its receptors are the best-characterized systems responsible for angiogenesis regulation in rheumatoid joints, making them great potential targets [45].

Among the cellular adhesion molecules, selectins are important in RA due to their role in the recruitment of leukocytes into synovial tissues [46]. Similar to the other targets mentioned above, selectins are also overexpressed in inflammatory cells and can be subdivided into *P*-selectins, E-selectins and L-selectins, according to the type of cells where they are expressed [46]. The E-selectins are upregulated in inflammation [46,47], and its blockage would be a useful strategy in RA treatment. Other therapeutic targets for RA therapy may include specific antigens differentially expressed on the surface of activated macrophages, such as CD163 [48], or components involved in immune cell activation, such as Bruton’s tyrosine kinase B that is involved in B cell activation [49]. Additional information of other targets may be consulted in [14,50].

## 4. Drug Delivery Nanosystems

Drug delivery systems appeared as a strategy to partially overcome the hindrances presented by conventional therapies, including the difficulty in crossing biological barriers and the incapacity of an active form of the therapeutic compound to achieve its target, either because of an early degradation or interaction with other molecules [51]. With drug delivery, it is possible to improve the pharmacokinetic and pharmacodynamic of a compound, decreasing the required dose and side effects, ultimately maximizing the therapeutic index [52].

Within drug delivery, nanosystems gained a major role as a therapeutic option, being currently explored for most of the pathologies. They can be divided into non-viral and viral vectors and, according to their properties, can be administered locally or systemically by several administration routes, such as intravenous (i.v.), intraperitoneal (i.p.); intra-articular (i.a.); intramuscular (i.m.); subcutaneous (s.c.); epicutaneous (e.c.); oral; ocular; nasal and transdermal administration.

From all the types of drug delivery nanosystems (nanoDDS), liposomes were the first that received market authorization in 1995, and currently are still a significant part of nanoDDS under investigation—being present in all stages of clinical development—and represent 20% of the nanoDDS in the market [53,54,55]. A detailed revision of liposomal formulations, either in clinical trials or in the market, can be consulted in Bulbake et al. [53].

### 4.1. Liposomal Formulations Developed for Rheumatoid Arthritis Treatment

New therapeutic strategies that use drug delivery nanosystems targeted to arthritic joints have been under investigation. In pathologies that affect a limited number of sites that are easily accessible, local administration routes play an important role [33,34]. In the case of RA, nanocarriers (e.g., liposomes, nanoparticles and hydrogels) have been administered by intra-articular (i.a.) route to decrease drug clearance and enhance patients compliance [50]. One of the latest examples is a conventional liposomal formulation that incorporates a prodrug of sulfapyridine, an active metabolite of sulfasalazine responsible for systemic side-effects [56]. This nanocarrier, upon i.a. administration on a complete Freund’s adjuvant-induced arthritis (CFA) rat model, demonstrated a significant reduction in the joint diameter, paw volume, pain threshold and in plasma and serum levels of biomarkers (IL-6, tumor necrosis factor-alpha (TNF-α), alkaline phosphatase (ALP), alanine transferase (ALT), aspartate aminotransferase (AST) and rheumatoid factor (RF) [57].

NanoDDS have also proven their efficacy as an optimized approach for systemic administration. These carriers can passively accumulate in arthritic joints through the EPR effect, enhancing drugs’ therapeutic effectiveness [58]. In addition to the passive targeting, successful delivery of nanosystems could be achieved via active targeting strategies. In this approach, functional and cellular changes that exist in arthritic joints are explored, for instance, through the targeting of macrophages, fibroblasts and angiogenesis [5,14].

Similar to the classification applied for conventional therapies, nanoDDS developed for RA treatment can also be subdivided into several classes: nonsteroidal anti-inflammatory drugs, glucocorticoids, disease-modifying antirheumatic drugs and biologic agent delivery nanosystems. Additionally, new molecules that are not included in the conventional therapies were explored in nanocarriers, such as compounds used in traditional Chinese medicine and natural compounds with reported anti-inflammatory effects [59,60]. For each of the previously mentioned classes, several types of nanoDDS were developed and evaluated in vitro and in vivo, displaying a therapeutic benefit in RA models. Due to the countless studies reported involving delivery nanosystems in RA and the major role of liposomes as an alternative therapeutic strategy—with a considerable presence in preclinical and clinical stages, as well as in the market—only this type of nanocarrier is exemplified in Table 1 and will be further discussed. Over the years, some reviews have been published either reporting several types of nanoDDS or focusing on a specific nanocarrier for RA treatment, including [33,50,61,62,63,64]. Concerning liposomal formulations, this manuscript reviews the data available until February 2021.

Several parameters are important for the characterization of liposomal systems, such as (i) the preparation method, (ii) the lipid composition, (iii) the ratio between the lipids and between the total lipid and the therapeutic compound used during the preparation, (iv) the mean diameter and polydispersity index (PDI), (v) the superficial charge (ζ), (vi) the incorporation/encapsulation efficiency (further referred as E.E.) and (vii) the loading capacity (L.C.). However, most of the studies only provide part of this information, namely the lipid composition with the respective ratio, the mean diameter and the E.E. obtained. As the last parameter is dependent on the initial concentration of drug—decreasing when drug concentrations are closer to the liposomal saturation limit—its use for comparison purposes is only adequate when the same drug-to-lipid ratio is used between different formulations. A more reliable factor in comparing nanocarriers is the loading capacity (µg drug/µmol lipid) obtained, but only a small number of studies present this information. For this reason, only the lipid composition, the respective molar ratio and the mean diameter are shown in Table 1. The other parameters will be further detailed in the description of the nanosystems when available.

Among the cases where E.E. is a useful parameter for liposomal evaluation are included studies, such as the one performed by Guimarães et al. [95] where the ethanol injection method was compared to the preconcentration method—a modified version of the former that was developed in this study—resulting in the enhancement of E.E. superior to 30%, without the requirement of the extrusion process, or the study of Srinath et al. [66] where four preparation methods were compared and, in each one, five different lipid compositions were evaluated. From the results obtained, the authors observed that the incorporation of indomethacin was higher in multilamellar vesicles (MLVs) than in large unilamellar vesicles (LUVs), with the thin-film hydration method being the one that resulted in the highest E.E. for all lipid compositions. Moreover, the inclusion of charged lipids, such as stearyl amine and phosphatidylglycerol, decreased in vitro drug release and reduced in vivo paw edema, resulting in a higher anti-inflammatory effect. Nonetheless, this study could be improved by: (i), including a complete characterization of liposomal formulations, such as the mean size of MLVs and LUVs; (ii) performing the comparison of E.E. in small unilamellar vesicles (SUVs)—whose therapeutic effect was assessed in RA models—instead of in MLVs and LUVs; (iii) avoiding the compartmentalized comparisons between liposomal formulations without, including the one with the highest E.E.

Table 1 shows that cholesterol is frequently used in formulations, followed by phosphatidylcholine and 1,2-distearoyl-*sn*-glycero-3-phosphoethanolamine-N-[methoxy (polyethylene glycol)-2000] (DSPE-PEG_2000_). This may be related to their role in liposomal formulations. Indeed, cholesterol can alter the packing of lipid chains, modulating membrane fluidity and permeability; phosphatidylcholine has a structural role and is one of the main constituents of biological membranes, and PEG-lipid conjugates confer long-circulating properties to liposomes [130]. In other studies, distinct lipids were used for a specific purpose, such as 1,2-dioleoyl-sn-glycero-3-phosphoethanolamine (DOPE) and cholesteryl hemisuccinate (CHEMS) due to their pH-sensitive properties, and RPR209120 cationic lipid for genetic material delivery [131].

Moreover, most nanocarriers from Table 1 display a mean diameter under 200 nm. This feature is generally used for i.v. administration of nanoDDS, the major administration route explored for RA, and enable nanocarriers accumulation in inflamed joints either by passive targeting, mainly when PEG-liposomes were used, or by active targeting when PEG-liposomes were functionalized with targeting agents. Additionally, when larger liposomal formulations were investigated, i.a. administration was the most employed, resulting in an enhanced anti-inflammatory effect [57,93,98]. Nevertheless, i.v. the administration was also described in studies where the effect of larger and smaller liposomes effect was compared, resulting in a higher therapeutic benefit of smaller nanocarriers, either when passive or active targeting strategies were applied [25,85].

#### 4.1.1. Liposomes Containing Nonsteroidal Anti-Inflammatory Drugs

From the division performed in Table 1, NSAID is the class of therapeutic agents that have been less investigated for liposomal drug delivery—only three distinct drugs were explored. This fact is possibly related to the minor role that NSAIDs have in RA from a therapeutic perspective: these drugs are incapable of protecting joints from damage; they only mitigate symptoms.

From the two liposomal formulations with indomethacin developed for systemic administration, one was studied in a carrageenan-induced rat paw edema model, where the inhibition of paw edema was observed [65], and in the other, indomethacin-loaded liposomes (E.E.: 28–46%) have proven to be more effective in reducing joint inflammation than free drug in both carrageenan-induced paw edema and Freund’s adjuvant arthritis rat models, as well as in reducing ulcers severity [66].

Intra-articular and transdermal administration routes also proved to be useful in RA treatment. In the first, a single i.a. injection of a gel formulation of diclofenac liposomes resulted in the reduction of joint swelling in antigen-induced arthritis (AIA) rabbit model [68], that has the advantage of being a chronic model of RA, where the destruction of cartilage and bone occurs, contrary to the carrageenan and Freund’s adjuvant arthritis models [132]. In the second, an emulsion containing diclofenac liposomes (E.E.: 14–23%) was topically administered, and ultrasounds were used to enhance skin permeation in a carrageenan-induced rat paw edema model, resulting in a high suppression of paw inflammation [69].

#### 4.1.2. Liposomes Containing Glucocorticoids

To improve their therapeutic effectiveness, glucocorticoids also have been included in nanosystems for distinct routes of administration. Within the i.a. route, liposomes incorporating triamcinolone acetonide-21-palmitate in their membrane showed enhanced drug retention in the articular cavity, as well as a reduction of the presence of inflammatory cells in the joints and a decrease in paw edema of arthritic rabbits when compared to the free drug [91]. Most studies reported for glucocorticoids in Table 1 were designed for i.v. administration, where dexamethasone was the most investigated drug for liposomal delivery—possibly due to its higher potency [133]—followed by prednisolone.

All the developed liposomal formulations of dexamethasone resulted in a higher therapeutic effect than free dexamethasone [79,82,83], being capable of a higher reduction in joint swelling, inflammation and destruction, even when a lower drug dose was used in liposomes [80,81]. Additionally, liposomal dexamethasone (PDI: ≤0.3, L.C.: 40 μg drug/μmol lipid) demonstrated its efficacy for longer periods, where free dexamethasone no longer presented a therapeutic effect, revealing its potential as a strategy to minimize administration frequency and avoid side-effects [81]. A similar dose-reduction effect was observed with prednisolone liposomes (L.C.: 58–75 μg drug/μmol lipid), where a single i.v. administration of liposomes caused remission of joint inflammation, along with a reduction of cartilage damage similar to the one obtained with multiple administrations of a ten times higher dose of free prednisolone, in two distinct models: murine collagen-induced arthritis (CIA) [72] and a rat AIA model [25]. Additionally, these liposomes were able to minimize bone erosion [70,71] and reverse the disease-induced weight loss [25,72]. Due to the promising therapeutic effect obtained with this nanocarrier, prednisolone PEG-liposomes have advanced to clinical trials (NIH identifiers indicated in Section 5.1).

Another strategy explored to further decrease the administered dose was the use of liposomes with glucocorticoid prodrugs that exhibit a lower clearance than the active form. In this case, two glucocorticoids—methylprednisolone hemisuccinate and betamethasone hemisuccinate—were evaluated in RA treatment. Liposomes encapsulating each of these two prodrugs (E.E.: 94%) resulted in a higher therapeutic effect than the free glucocorticoids in an AIA rat model, even when liposomes were injected with lower doses [74]. Moreover, a significant reduction in RA severity throughout early and late disease stages was observed. In another study, the effect of these two nanoformulations, upon i.v. or s.c. The administration was compared to weekly or daily treatment with the free drugs or with two biological DMARDs—infliximab and etanercept. The results obtained showed that liposomes of both prodrugs (E.E: >90%, drug-to-lipid molar ratio: >0.35) significantly suppressed arthritis in an AIA rat model, reducing the arthritis score and inhibiting the production of proinflammatory cytokines, either compared to higher doses of the free drugs or biologic DMARDs [75].

Besides the passive targeting strategy previously described for glucocorticoid delivery, active targeting approaches have also been investigated for RA treatment, with systemic delivery of liposomes. In the case of dexamethasone, five distinct targeting agents were used, namely sialic acid, folate, mannose, RGD peptide and ART-2 lipopeptide (Table 1). The last two agents target nanocarriers for endothelial cells in the blood vessels at the inflamed synovium, whereas folate and mannose target them to macrophages, and sialic acid targets them to peripheral blood neutrophils. Overall, all the liposomal formulations demonstrated a therapeutic benefit in comparison to the non-targeted liposomes or free drugs. In dexamethasone nanocarriers targeted with RGD (PDI: 0.1, E.E.: 3–6%; L.C.: 30–60 μg drug/μmol lipid) or ART-2 (PDI: 0.3, ζ: 40 mV, E.E.: 73–78%) peptides, a prolonged anti-inflammatory effect was observed in arthritic rats [20], resulting in an enhanced efficacy without increasing the adverse side-effects [89]. Sialic acid-targeting resulted in the inhibition of RA progression by dexamethasone liposomes (PDI: 0.2, ζ: −16 to −21 mV, E.E.: 90–95%), with a decrease in proinflammatory cytokines and transaminase levels [84]. Interestingly, the effect of three distinct sizes of liposomes (300 nm, 150 nm and 75 nm—PDI: <0.2, ζ: −39 mV, E.E.: 97–98%) was compared, and the smallest liposomes were the ones that resulted in the higher suppression of paw thickness and reduction of arthritis scores, proinflammatory cytokines and transaminase levels [85]. Nonetheless, arthritic rats treated with all the sialic acid-targeted dexamethasone formulations exhibited a significant reduction in joint injury and pathological score in comparison to the free drug [85].

A distinct approach was used with dexamethasone liposomes targeted with folate, where the effect of a combined therapy among liposomes, microbubbles and ultrasounds was assessed [88]. This study demonstrated that folate-targeted liposomes (ζ: −3 mV, E.E.: 9–10%) resulted in a therapeutic benefit when compared to non-targeted liposomes and free dexamethasone. However, the therapeutic effect of folate-targeted liposomal dexamethasone was even higher when the treatment included the destruction of microbubbles with ultrasounds, demonstrating a synergistic effect. The combination of both strategies resulted in a greater decrease in inflammatory cytokines; an inhibition of joint swelling, with reduced joint synovial hyperplasia and infiltration of inflammatory cells; and in the protection against cartilage damage and bone erosion in a rat CIA model [88].

With prednisolone liposomes, only one of the three targeting agents used was common to the ones used with dexamethasone—RGD peptide—the others were HAP-1 peptide and hyaluronic acid that targeted liposomes to fibroblast-derived type B synoviocytes or to synovial cells, respectively. The targeting of prednisolone liposomes also improved disease outcomes after the treatment compared to non-targeted liposomes [78]. In the study of Vanniasinghe et al. [78], a direct comparison of the targeting effect of RGD and HAP-1 in prednisolone liposomes (PDI: <0.4, ζ: −16.2 to −24.0 mV) was performed, where it was possible to observe that, despite both being capable of decreasing RA severity in an AIA rat model, HAP-1 resulted in a higher survival rate and enable a decrease in the necessary dose for a therapeutic effect, highlighting its superior potential as a targeting agent in RA treatment.

Besides comparing targeting agents, comparison of nanocarriers with distinct drugs is also crucial since they enable the direct evaluation of which one has the most potential to be a good alternative in RA therapy. Despite their utility, these types of studies are not common. An example is a comparative study performed to access the therapeutic efficacy of liposomes with three distinct glucocorticoids (L.C.: 58–75 µg drug/µmol lipid), namely prednisolone disodium phosphate, dexamethasone disodium phosphate and budesonide disodium phosphate [73]. The authors observed that among the three, budesonide liposomes were the most promising candidates for glucocorticoid liposomal formulations since they induced a full remission of clinical arthritis signs in a shorter time, with a lower dosage and with minimum adverse side-effects.

#### 4.1.3. Liposomes Containing Disease-Modifying Antirheumatic Drugs

Within DMARDs, methotrexate has been the most studied drug for liposomal delivery, either using passive or active targeting strategies. The first attempt to encapsulate this drug in liposomes was made in 1988 by Foong and Green for i.a. administration in arthritis rabbits [92]. A higher accumulation of methotrexate at the joint was obtained, and the formulation (L.C.: 10 µg drug/µmol lipid) was able to suppress arthritis development if injected at the time of disease induction, even at a lower dose, comparatively to the free drug. However, neither free nor liposomal DMARD were able to suppress established arthritis [93]. Later, the i.v. administration of methotrexate liposomes was also studied in a CIA rat model, where an improved anti-inflammatory activity, with a decrease in paw edema and arthritis score, was observed in the short-term for conventional liposomes and in the long-term for PEG-liposomes [96,97].

Active targeting of methotrexate liposomes was performed with folate, mannose and iRDG peptide. In the first, an i.p. administration of these liposomes in a murine CIA model caused a higher accumulation in the inflamed joints with an increased internalization in activated macrophages, being capable of avoiding the development of arthritis when applied as a prophylactic treatment, in contrary to the non-targeted formulation or free methotrexate [101]. In iRGD active targeting, the combination of echogenic liposomes (PDI: 0.2, ζ: −6 to −14 mV, E.E.: 69%) and ultrasounds were explored, resulting in dexamethasone release from liposomes induced by low-frequency ultrasounds [103]. In this study, a higher therapeutic effect was obtained when liposomes and ultrasounds were combined as a therapy, in comparison with all the other groups—free drug, targeted liposomes without ultrasounds and non-targeted liposomes with ultrasounds. The reduction of arthritis score and inflammatory cell infiltration, along with the absence of cartilage and bone destruction, were preferentially observed when the combinatory therapy was applied in a murine CIA model. Additionally, the simultaneous inclusion of indocyanine green in iRGD-targeted dexamethasone liposomes enabled near-infrared fluorescence imaging, demonstrating the potential of this nanocarrier as a theranostic agent.

Another nanoDDS with triggered methotrexate release is a folate-targeted liposomal formulation (PDI: 0.2, ζ: −4 mV, E.E.: 84–89%) that co-encapsulated methotrexate and catalase, an enzyme that converts hydrogen peroxide in oxygen and water. Since high levels of intracellular ROS are observed in activated macrophages existent in RA, when liposomes are internalized by these cells, oxygen is produced, leading to the destruction of the liposomal membrane with a consequent release of methotrexate [104]. This system resulted in the enhancement of the therapeutic efficacy with minimal toxicity and maintenance of body weight when compared to free or liposomal methotrexate and to non-targeted liposomes with both compounds in a CIA rat model.

The investigation in targeted delivery nanosystems for biologic DMARDs is only in the beginning, with minimal numbers of nanoDDS developed. The only example reported in Table 1 is tocilizumab encapsulated in liposomes (PDI: 0.2, ζ: −2 mV, E.E.: 86%) that, upon i.v. administration demonstrated an improved therapeutic effect in comparison with free tocilizumab, with a higher reduction in paw edema, arthritis joint score, proinflammatory cytokine expression, and bone erosion in arthritic rats [105].

#### 4.1.4. Liposomes Containing Biologic Agents

The first biologic agents used in therapy were proteins, such as superoxide dismutase (SOD), an enzyme with an anti-inflammatory activity that catalyzes the dismutation of superoxide radicals (O_2_^•−^) to molecular oxygen (O_2_) and hydrogen peroxide (H_2_O_2_) [134]. This enzyme was first encapsulated in liposomes in 1985, and a higher therapeutic effect was achieved with reduced toxicity in RA [110]. Later, SOD was encapsulated in distinct liposomal formulations (PDI: <0.4, L.C.: 12–15 µg SOD/µmol lipid), and it was possible to observe that after i.v. [108] or s.c. [106] administration in an AIA rat model, smaller PEG-liposomes (PDI: <0.1, L.C.: 12–15 µg SOD/µmol lipid) were more efficient in delivering SOD to arthritic sites, resulting in a concomitant stronger paw edema reduction.

In another study, the effect of SOD localization in liposomes was evaluated by comparing conventional SOD that was encapsulated in liposomes with an acylated SOD that was incorporated in the liposomal membrane. Liposomes with acylated SOD (PDI: <0.2, L.C.: 3–9 µg SOD/µmol lipid) showed a faster onset of anti-inflammatory activity, possibly because there is no need to release the enzyme since it is partially exposed at liposome surface [107]. In 2015, a different approach was studied, where SOD was covalently linked to the surface of long-circulating liposomes (L.C.: 50–60 µg SOD/µmol lipid). After i.v. administration in an AIA rat model, this formulation showed higher anti-inflammatory activity than liposomes with encapsulated SOD, demonstrating that not only the inclusion of drugs in nanocarriers but also their location in them could have a significant impact on the therapeutic outcome [109].

Besides enzymes, other proteins, such as human lactoferrin, cytokines and antibodies, were also explored as a therapeutic strategy in RA [111,115,116]. An example is a liposomal formulation containing a cytokine capable of inducing apoptosis, named Apo2 ligand or tumor necrosis factor-related apoptosis-inducing ligand (Apo2L/TRAIL), that was injected i.a. in the inflamed joint space in an AIA rabbit model [111]. In this study, a reduction of synovial hyperplasia until nearly the normal value was obtained and joint inflammation was reduced by 60% after liposomal treatment, compared to a 30% decrease with the free cytokine.

Active targeting strategies have also been applied in liposomes with cytokines. In this case, IL-27 liposomes (PDI: 0.1, ζ: 19–37 mV, E.E.: 38–41%) were targeted with ART-1 lipopeptide to synovial endothelial cells [117]. This nanocarrier resulted in higher suppression of RA progression in an AIA rat model that non-targeted IL-27 liposomes or free IL-27, with a significant decrease in transaminases levels, cartilage damage and bone erosion.

A distinct approach, which also involves cytokines, is a liposomal formulation containing gold nanoparticles and with anti-IL-23 antibody covalently linked to the liposomal surface (PDI: 0.1, ζ: −24 mV, E.E.: 74–90%). This system enables the capture and inactivation of IL-23, a proinflammatory cytokine, potentially attenuating inflammation and immune cell recruitment in RA [116].

Further to the use of proteins, the therapeutic effect of genetic material is also significantly improved with the use of nanocarriers since when used alone, they present a short biological half-life [135,136]. In the last decade, gene therapy has been successfully translated to clinics in other pathologies, which motivated more investigation in this area. In RA, silencing RNA was investigated upon i.v. administration in a murine CIA model, namely through liposomal formulations encapsulating siRNA for TNF-α (ζ: 30–40 mV) or for IL-1β, IL-6 or IL-18, individually. The former allowed a complete inhibition of experimentally induced arthritis with a decreased TNF-α secretion by 50–70% [112]; the latter delayed RA onset and progression through the inhibition of joint swelling and bone destruction for siRNA of all interleukins used individually. Using simultaneously formulations of the three interleukins (1β, 6 and 18) siRNAs resulted in a notable therapeutic effect, where was achieved a decrease in inflammation and joint destruction comparable to the obtained with TNF-α siRNA liposomes [113]. More recently, gene silencing has been explored by targeting gene regulators, such as microRNAs, that also benefit from their inclusion in nanoDDS. One example of microRNA that has been explored in RA treatment is a liposomal formulation (PDI: 0.1, ζ: 30 mV) that contained a complex between microRNA-23a and polyethylenimine [114]. With this system, it was possible to observe a reduction of paw edema, cartilage degradation and bone damage in comparison to non-treated animals in an AIA rat model. Additionally, the infiltration of inflammatory cells in joints and proinflammatory cytokines expression also decreased, while the RA-induced loss of body weight was partially recovered, demonstrating a therapeutic benefit.

#### 4.1.5. Liposomes Containing a Combination of Distinct Therapeutic Compounds

Besides the use of a therapeutic agent by itself in liposomes, some studies investigated the effect of a combination of drugs from distinct classes. In these more complex systems, active targeting with folate was used as a strategy to enhance even more the treatment outcome. For instance, a glucocorticoid and a DMARD, namely prednisolone and methotrexate, were simultaneously loaded in a liposomal formulation (PDI: 0.1, ζ: 8 mV, E.E.: 62–71% for prednisolone and 44–47% for methotrexate) targeted with folate and their effect after i.v. administration in a rat model of RA was assessed [118]. In comparison to a mixture of individual free drugs and to non-targeted liposomes with both drugs, the active targeting of liposomes with folate resulted in a higher drug concentration in joints and consequently in the highest inhibition of paw edema, demonstrating a greater therapeutic potential.

In another study, a more complex carrier was developed, namely folate-targeted liposomes (ζ: −24 mV) co-encapsulating methotrexate and calcium phosphate nanoparticles that contained p65 siRNA [119]. In this study, targeted liposomes with the DMARD and siRNA demonstrated a superior effect in reducing paw edema and arthritis score in a murine CIA model than the groups that were i.v. injected with naked p65 siRNA, free methotrexate or non-targeted liposomes containing both compounds. Interestingly, while free methotrexate was able to attenuate paw edema, naked siRNA was not able to produce any therapeutic benefit, demonstrating the impact that nanosystems have in genetic material delivery.

Dexamethasone was also evaluated in a very complex nanoDDS, composed of folate-targeted liposomes that incorporated dexamethasone and co-encapsulated nuclear factor-κB (NF-κB) decoy oligodeoxynucleotides (ODNs) and gold nanorods (GNRs) (PDI: 0.2, ζ: −13 mV, E.E.: 50% for dexamethasone and 36% for ODNs/GNRs) [120]. This carrier was designed to have a triple therapeutic effect in RA that culminates in the inhibition of the NF-κB inflammatory pathway. The individual contribution of each component was the following: (i) dexamethasone exerted the anti-arthritic effect previously described for corticosteroids in Section 2; (ii) ODNs inhibited the interaction of p50/p65 proteins with inflammatory gene sequences, partially inhibiting the NF-κB pathway; and (iii) GNRs released heat upon near-infrared laser irradiation, accelerating the destruction of liposomes, with the consequent release of the therapeutic compounds. Additionally, authors have hypothesized that GNRs could also play a role in reducing the signaling of Toll-like receptor-4 (TLR-4) and TLR-9, decreasing NF-κB signaling and subsequently reducing inflammation. In a murine AIA model, the i.v. injection of targeted liposomal formulation combined with laser irradiation resulted in the most significant decrease of paw edema, arthritis score and inflammatory cells infiltration [120]. Moreover, proinflammatory cytokine levels were also reduced and enhanced cartilage protection was observed with this treatment in comparison with all the other groups—free dexamethasone; folate-targeted liposomes with dexamethasone; the mixture of ODNs and GNRs with laser irradiation; folate targeted liposomes with GNRs and ODNs combined with laser irradiation; and non-targeted liposomes with the three compounds—demonstrating the potential of combined approaches in RA therapy.

#### 4.1.6. Liposomes Containing Nonconventional Compounds

Some compounds that do not fall into the four main classes of therapeutic agents used in RA treatment, such as natural compounds and products used in traditional Chinese medicine, have also been investigated. An example is a thermosensitive liposomal formulation (PDI: 0.2, ζ: −4 mV, E.E.: 95–98%) with sinomenine hydrochloride that is released after microwave hyperthermia [59]. A temperature surge (localized hyperthermia) was then used as a trigger for sinomenine release from liposomes leading to a higher therapeutic effect when compared to free sinomenine or to hyperthermia alone. In this study, the higher therapeutic effect was translated in the reduction of paw edema and arthritis scores, along with decreased proinflammatory cytokine levels, synovial inflammation and bone erosion.

Part of the research involving new compounds also takes advantage of active targeting to further enhance the treatment outcome. Such examples are conventional mannose-targeted liposomes incorporating morin, *p*-coumaric acid or withaferin-A and PEG-liposomes containing core peptide, targeted with RGD or HAP-1 peptides—see Table 1 [78,86,87,102]. All these nanocarriers demonstrated an improved therapeutic benefit in an AIA rat model, where the following effects were observed: (i) reduction of paw edema; (ii) downregulation of proinflammatory cytokines; (iii) suppression of inflammatory cells infiltration; (iv) minimization of cartilage damage and (v) decreased or inexistent bone erosion. Additionally, some other factors were investigated in some of the studies, including the survival probability that was increased in the case of core peptide liposomes targeted with RGD or HAP-1; the production of ROS and nitric oxide and the RA-induced loss of body weight that decreased upon treatment with mannose-targeted liposomes with morin, *p*-coumaric acid or withaferin-A. Interestingly, a comparison among some of these nanoDDS with liposomes containing a conventional drug was also performed, where it was observed that mannose-targeted liposomes that contained morin (ζ: −54 mV, E.E.: 83–90%) exerted a similar therapeutic effect than the ones containing dexamethasone, with the exception of joint damage, that was further reduced with the morin formulation [86]. Mannose-targeted liposomes with incorporated withaferin-A (PDI: 0.1, ζ: −49 mV) were also compared to ones containing dexamethasone, and the results demonstrated that they have similar effects, except for paw edema, cartilage damage and anti-inflammatory marker expression, where liposomal withaferin-A caused a higher reduction in the first two and a higher expression of the third [87].

Another example is the mannose-targeted liposomal formulation with *p*-coumaric acid (PDI: 0.1, ζ: −56 mV, E.E.: 95%) that was compared to similar liposomes containing methotrexate. Generally, the therapeutic effect was similar, but animals showed greater balance on the beam walk test when treated with *p*-coumaric acid liposomes [102]. A direct comparison was also performed with liposomes containing core peptide (PDI: <0.4, ζ: −16 to −24 mV) or prednisolone, targeted with RGD or HAP-1 peptides. In this study [78], the liposomal formulation with *p*-coumaric acid and HAP-1 peptide demonstrated the highest therapeutic effectiveness in RA since it enabled a dose-reduction combined with significant and long-term suppression of the inflammation in arthritic rats. Moreover, the targeting with HAP-1 peptide demonstrated better results than RDG when used with both compounds, allowing their dose reduction. From these two results is possible to conclude that HAP-1 targeting appears to be a better targeting agent from RA and that core peptide could be a good possibility for liposomal drug delivery in RA treatment.

The previously presented systems for nonconventional compounds were studied upon i.v. administration. However, other routes have also been investigated, namely transdermal and oral, that usually have improved patient compliance in comparison to injectable routes. The transdermal route has been used to administer liposomes (E.E.: 88%) with triptolide—a compound used in traditional Chinese medicine for RA—that were loaded into a hydrogel patch [60]. The systemic delivery of these liposomes was enhanced using a micro-needle array that promoted transdermal absorption. The three liposomal triptolide doses evaluated were able to reduce joint swelling and decrease proinflammatory cytokines in a dose-dependent manner, but all resulted in an improved therapeutic outcome in comparison to non-treated arthritic rats.

Oral administration was explored to deliver coenzyme Q10—an antioxidant used in dietary supplements—in a hybrid system involving liposomes and gold particles [129]. Treatment of a murine CIA model with this nanocarrier resulted in a higher decrease in proinflammatory cytokines, cartilage damage and bone erosion in comparison to oral Q10, demonstrating the therapeutic potential that known dietary supplements could exert when properly formulated.

## 5. Translation to the Clinic

### 5.1. Clinical Trials with Drug Delivery Nanosystems in Rheumatoid Arthritis

Many clinical trials involving RA are already in development. A search for RA treatments at the USA National Library of Medicine database (https://www.clinicaltrials.gov, accessed on 17 February 2021) shows 2595 clinical trials, and the European Union Clinical Trials Register (https://www.clinicaltrialsregister.eu/, accessed on 17 February 2021) shows 798 clinical trials, either finished or still ongoing. Most of these clinical trials, especially the ones in phases III and IV, include either synthetic or biological drugs. In both databases were only reported seven studies involving drug delivery nanosystems, as indicated in Table 2.

The percentage of nanoDDS in the clinical trials identified for RA treatment is currently less than 1%, clearly demonstrating that there is still a long way before the successful clinical translation of nanoDDS for this pathology, with the focus still remaining on conventional or biologic drugs. Nevertheless, in the last decade, several gene therapy products have entered the market [137], reinforcing the investigation on nanoDDS for gene therapy that resulted in active clinical trials with viral vectors for RA treatment. Furthermore, it is possible to observe that the trials in the most advanced phases are liposomal formulations, indicating that more recent technologies could be facing difficulties in moving beyond the preclinical stages.

### 5.2. Transition of Drug-Delivery Nanosystems to the Market

With nanoDDS is possible to enhance compounds therapeutic index, either through the improvement of drugs pharmacokinetic or pharmacodynamic properties (e.g., carriers protect drugs from early degradation and enable them to cross biologic barriers more easily); the direct delivery to a specific target and the minimization of severe side-effects [51,52]. If properly applied, delivery systems have the potential to exert a major impact on human health. Nonetheless, there are only a limited number of nanocarriers in clinical trials and, even fewer, in the market. This fact highlights the discrepancy existent between nanoDDS with promising results in preclinical stages and the ones that demonstrated therapeutic potential in the clinical setting [138]. Indeed, currently, no nanoDDS received market approval for RA treatment, despite all the encouraging results reported in Section 4.

Clear identification of the main factors responsible for the inefficient clinical translation of nanoDDSs must be performed to avoid the same errors, improving their translation to successful clinical trials and, ultimately, to the market. Such obstacles prevent the full impact and translation of nanoDDS into clinically feasible therapies, among which are the difficulties in the preclinical characterization of nanosystems, in their scale-up production to an industrial level and the evaluation of their in vivo pharmacokinetics and pharmacodynamics properties. Additionally, safety evaluation could be more difficult with these new carriers, and, frequently, they are not developed in compliance with good manufacturing practice (GMP) regulations [55,139]. The slowness of the process itself and the high-cost for the pharmaceutical industry also hinder nanosystem clinical translation. The latter is even more important in the case where nanoDDS are used with approved drugs—which is the case of most of the currently marketed formulations—since the industry would possibly invest even more than in the original drug approval process because the required techniques are frequently more complex and is necessary to repeat all the studies (e.g., efficacy and toxicity) for nanocarriers approval, due to their distinct pharmacokinetic and biodistribution profiles from the original drugs [51,140]. The inexistence of guidelines specific for nanosystems that are equivalent for all the regulatory agencies is another critical factor that negatively affects market authorizations, especially for products that combine several therapies and/or technologies, such as theranostic agents [141,142]. A more detailed description of the aforementioned factors can be consulted in Germain et al. [55] and Taha et al. [143].

Taking all these factors into consideration during the development stages of nanoDDS may potentially result in improved clinical translation. An example is the development of systems following GMP guidelines to guarantee consistent quality, using biocompatible starting materials and techniques that allow robust and scalable manufacturing to facilitate the commercialization process [139,143]. Moreover, the new therapy should be compared with the gold standard used in the clinic [138,141]. In RA, the treatment regimen is highly dependent on the patient’s reaction to each therapy, and, frequently, drugs from several classes are used simultaneously, which hamper the proper evaluation of a nanocarrier therapeutic value.

Nanocarrier complexity is another barrier that has slowed their translational success. For this reason, investigators should avoid highly complex designs, such as the one described by Xue et al. [120], when developing nanoDDS, since it will simplify the manufacture and characterization processes, improving the large-scale reproducibly and reducing the final product cost [144,145].

The use of adequate in vitro and in vivo models that more closely resembles the human physiopathology of the disease can improve predictions of the therapeutic outcome in humans, especially in the case of chronic diseases [138]. In RA, model selection should be carefully considered due to the existence of several models that display distinct characteristics **[132].** An example of the importance of the in vivo model choice was reported by Hua et al. Hu [146] in a study with liposomal loperamide that, initially was evaluated in a CFA model demonstrating the promising anti-inflammatory activity, but afterward was evaluated in an AIA model—that is more complex and presents more similarity to human RA—resulting in an enhanced severity of inflammation and in the acceleration of arthritis progression.

Studies where RA treatment was evaluated using different nanoDDS with the same therapeutic agent or using distinct drugs in the same nanocarrier—as performed in [73] and [147], respectively—should also be recommended, since it would enable the direct comparison of the therapeutic benefit and the definition of the most promising characteristics for each drug and delivery system.

Moreover, during initial study planning, treatment schedules should be carefully planned considering the disease specificities. For instance, prophylactic treatment or treatment where the therapy starts shortly after RA onset will hardly produce useful results because RA is not possible to predict, and there is an interval between the beginning of symptoms, the adequate diagnostic, and the medical treatment. The administration route used in the studies also has an important role, since when possible, patient compliance should be maximized (e.g., by avoiding injectable routes), and the use of large injection volumes should be avoided—it would imply increasing medical costs, decreasing the cost-effectiveness of the nanocarrier [143].

In inflammatory diseases, it is of utmost importance to consider its specificity in terms of the inflammatory profile, a factor that is lacking in many studies and is partially responsible for the insufficient preclinical success and clinical translation. In RA, despite joints being the principal affected area, inflammatory mediators are systemically transported. Due to this characteristic, using exclusively an active targeting approach or a carrier that only exhibited a local effect is not the most advisable since it would result in a limited therapeutic effect. In this case, developing a nanoDDS—either alone or as a combined therapy—that could tackle the local and systemic inflammation would be preferable. Additionally, since RA is a chronic inflammatory process where inflammation is continuously perpetuated, a system that enabled a more prolonged therapeutic effect would be desirable.

Besides all these aspects, the collaboration between academia, clinicians, experts from all stages of pharmaceutical development and regulatory authorities must continue to create standardized protocols and uniform regulations worldwide that will increase the number of nanoDDS successfully translated into the market [145,148].

## 6. Conclusions

From the wide panoply of inflammatory diseases, the chronic type is a major concern since it is associated with the permanent disability of patients and to severe socioeconomic problems due to the high costs, both for therapy and care. A special focus should be attributed to RA due to its large worldwide prevalence. Despite the multiple therapies available for this pathology, currently, there is no cure, and each therapy presents side-effects that are very important due to long-term use. NanoDDS, such as liposomes, enclose a great therapeutic potential due to their ability to minimize side effects and to enable a specific delivery to a target site and a controlled drug release. However, the encouraging results obtained with multiple carriers in preclinical studies only resulted in three clinical trials with liposomal formulations and four with viral vectors and no market approval granted to any treatment. The failure in translating the success obtained in preclinical studies to clinical trials due to the inefficient active targeting effect in humans and the lack of specific and uniform regulations are the factors that should be addressed to enhance nanoDDS clinical translation. However, since these drawbacks have been surpassed in other diseases, the same will potentially occur, resulting in nanocarriers’ approval for RA treatment in the clinic.

## Figures and Tables

**Table 1:** Examples of liposomes developed for rheumatoid arthritis treatment divided according to the therapeutic agent used. The lipid composition, the corresponding molar ratio and the mean diameter were indicated for each liposomal formulation.

Therapeutic Agent	Drug Delivery Nanosystems Developed	Lipid Composition (Molar Ratio)	Diameter (nm)	Reference
Nonsteroidal Anti-inflammatory Drug	Liposomes with incorporated indomethacin	SL:Chol:SA/DCP (7:3:1)	n.r.	[65]
		EPC:Chol:SA/PG (1:0.5:0.1/0.2)	50 or 100	[66]
	Liposomes with incorporated celecoxib	Lipova E120:Chol: DSPE-PEG_2000_ (9:1:0.25)	92	[67]
	Gel formulation of liposomes with encapsulated diclofenac sodium	DMPC:Chol:DCP (7:1:2)	235	[68]
	Oil/water emulsion of liposomes with incorporated diclofenac	EPC:DCP (9:1 or 7:3)	4430–5400	[69]
		EPC:Chol (9:1 or 7:3)	3590–4280	
Glucocorticoid	Liposomes with encapsulated prednisolone phosphate ^1^	DPPC:Chol:DSPE-PEG_2000_ (1.85:1:0.15)	90–110 or 450–500	[25,70,71,72,73]
	Liposomes with encapsulated methyl prednisolone hemisuccinate	HSPC:Chol:DSPE-PEG_2000_ (55:40:5 or 54:41:5)	68–98	[74,75]
	Poly-(hydroxyethyl L-asparagine) (PHEA)-liposomes with encapsulated prednisolone phosphate	DPPC:Chol:PHEA-DODASuc (1.85:1.0:0.15)	144–148	[76]
	pH-sensitive liposomes with incorporated prednisolone, targeted with hyaluronic acid	DPPE:CHEMS (6.5:3.5)	113–119	[77]
	Liposomes with encapsulated prednisolone phosphate, targeted with RGD or HAP-1 peptides	DPPC:Chol:DSPE-PEG_2000_: DSPE-PEG_2000_-Mal (1.85:1.0:0.075:0.075)	95–105	[78]
	Liposomes with encapsulated dexamethasone phosphate	DPPC:Chol:DSPE-PEG_2000_ (1.85:1.0:0.15)	90–100	[73]
		DPPC:DPPG:Chol (50:10:40)	280–310	[79,80,81]
	Liposomes with incorporated dexamethasone	SPC:Solutol HS 15 (3:1)	60	[82]
	Polymerized liposomes with incorporated dexamethasone	DC_8,9_PC:DSPE-PEG_2000_ (9:1)	112–131	[83]
	Liposomes with incorporated dexamethasone palmitate, targeted with sialic acid	HSPC:Chol (55:40)	130–138	[84]
		DSPC:DSPG:Chol (8.9:2.4:1)	71–79, 146–154 or 295–305	[85]
	Liposomes with incorporated dexamethasone palmitate, targeted with mannose	DSPC:Chol (60:35 or 60:32.5)	142–146 or 176–190	[86,87]
	Liposomes with encapsulated dexamethasone sodium phosphate, targeted with folate (FA)	DPPC:Chol:DSPE-PEG_2000_-FA (64:30:5)	157–159	[88]
	Liposomes with encapsulated dexamethasone, targeted with RGD peptide	DPPC:Chol:DSPE-PEG_2000_: DSPE-PEG_2000_-Mal (1.85:1:0.075:0.075)	100	[20]
	Liposomes with encapsulated dexamethasone, targeted with ART-2 lipopeptide	DOPC:DOPE:Chol: DSPE-PEG_2000_-NH_2_ (1:0.6:0.4:0.05)	96–105	[89]
	Liposomes with encapsulated betamethasone hemisuccinate	HSPC:Chol:DSPE-PEG_2000_ (55:40:5 or 54:41:5)	68–98	[74,75]
	Liposomes with encapsulated betamethasone, targeted with folate	DSPC:Chol:DSPE-PEG_2000_: DSPE-PEG_3400_-FA (56:40:4:0.1)	90–110	[90]
	Liposomes with encapsulated budesonide phosphate	DPPC:Chol:DSPE-PEG_2000_ (1.85:1:0.15)	90–100	[73]
	Liposomes with incorporated triamcinolone acetonide	DPPC:Chol:PA (8:3:1)	n.r.	[91]
Disease-modifying Antirheumatic Drug	Liposomes with encapsulated methotrexate sodium salt	EPC:Chol:DCP (5:5:1)	1070	[92,93]
		EL:Chol:PA (7:2:1)	100	[94]
		DOPE/EPC:Chol:DSPE-PEG_2000_ (54:36:10)	121–136/194–208	[95]
	Liposomes with incorporated methotrexate	EPC:Chol:PA (7:2:1) or DSPC:Chol:DSPE-PEG_2000_ (10:5:1)	100	[96,97]
		EL:Chol:PA (7:2:1)	100 or 1200	[98,99]
		POPC:Chol:DMPA	1200	[100]
	Liposomes with encapsulated methotrexate, targeted with folate	DOPE:Chol:DSPE-PEG_2000_-CA (n.r.)	120	[101]
	Liposomes with incorporated methotrexate, targeted with mannose	DSPC:Chol (60:35)	122–127	[102]
	Echogenic liposomes containing methotrexate and indocyanine green, targeted with iRGD peptide	DPPC:Chol:DSPE-PEG_2000_: DSPE-PEG_2000_-Mal (n.r.)	109–117	[103]
	Liposomes with co-encapsulated methotrexate and catalase, targeted with folate	POPC:Chol:S100-FA (13.2:1.9:0.6)	141–150	[104]
	Liposomes with encapsulated tofacitinib citrate	SPC:Chol (1:1)	55–63	[105]
	Liposomes with incorporated sulfapyridine or an amide prodrug of sulfapyridine	P-90G:Chol (6.3:3.1 or 5.5:4.7)	455–470 or 762–930	[57]
Biologic Agent	Liposomes with encapsulated or covalently linked superoxide dismutase	EPC:Chol:SA (7:2:1)	90, 110 or 210	[106,107,108,109]
		EPC:Chol:DSPE-PEG_2000_ (1.85:1:0.15)	90–110, 200 or 450	
		EPC:Chol:DSPE-PEG_2000_: DSPE-PEG_2000_-Mal (68.25:30.5:0.5:0.75)	120	
		n.r.	n.r.	[110]
	Liposomes linked to tumor necrosis factor-related apoptosis-inducing ligand (Apo2L/TRAIL)	EPC:SM:Chol:DGS-NTA (7.1:3.9:2.6:0.5)	150–200	[111]
	Liposomes encapsulating siRNA for TNF-α, IL-1β, IL-6 or IL-18	DOPE:RPR209120:carrier DNA (n.r.)	1500–2000	[112,113]
	Liposomes containing miR-23a/polyethylenimine (PEI) complex	DSPC:DSPE-PEG_2000_ (n.r.)	104–109	[114]
	Liposomes encapsulating human lactoferrin	DPPE:Chol:SA (5:5:1)	200	[115]
	Liposomes with anti-IL-23 antibody covalently linked to the surface, containing gold nanoparticles	EPC:Chol:DSPE-PEG_2000_-Mal (0.85:1:0.15)	127–133	[116]
	Liposomes with encapsulated IL-27, targeted with ART-1 lipopeptide	DOPC:DOPE:Chol: DSPEPEG_2000_-NH_2_ (1:0.5:0.5:0.01)	92–95	[117]
Combination of Therapeutic Compounds from Different Classes	Double liposomes with encapsulated prednisolone and incorporated methotrexate, targeted with folate	inner liposomes: DSPC:Chol:SA (7.5:2.5:0.5)/outer layer: DSPC:Chol:DSPE-PEG_2000_-FA (n.r.)	157–160/426–433	[118]
	Liposomes with co-encapsulated methotrexate and calcium phosphate nanoparticles that contained p65 siRNA, targeted with folate	DSPC:Chol:DSPE-PEG_2000_: DSPE-PEG_2000_-FA (4:1.2:0.15:0.04)	170	[119]
	Liposomes with incorporated dexamethasone and co-encapsulated nuclear factor-κB (NF-κB) decoy oligodeoxynucleotides and gold nanorods, targeted with folate	Lipoid E80:Chol:DSPE-PEG_2000_-FA (6.4:2.6:0.03)	95–113	[120]
Non-conventional Compound	Liposomes with incorporated berberine	DSPC:Chol:DSPE-PEG_2000_ (60:35:2.5)	157–161	[114]
	Liposomes with incorporated dimethyl curcumin	SPC:Chol (1.3:2.6)	<200	[121]
	Liposomes with encapsulated clodronate	PEG_400_-S:Chol:SDS (1.8:1.8:0.45)	858–942	[122]
		EPC:Chol:DPPA (7:7:1)	100	[123,124]
		EPC:Chol (2:1)	n.r.	
		EPC:Chol (n.r.)	120–160	[125]
		DSPC:DSPG:Chol (n.r)	n.r.	[126]
	Thermosensitive liposomes with encapsulated sinomenine hydrochloride	DPPC:SPC:Chol (5.1:1.6:0.7)	111–121	[59]
	Hydrogel patch containing liposomes with incorporated triptolide	EL:Chol (2.9:1.2)	183–220	[60]
	Dimeric artesunate phospholipid-conjugated liposomes	Di-ART-GPC	70–83	[127]
	Liposomes with incorporated naringin and encapsulated sulforaphane or phenethyl isothiocyanate	DPPC:Chol:DSPE-PEG_2000_ (15:4:1)	147–159	[128]
	Liposome/gold hybrid nanoparticles containing coenzyme Q10	DSPC (n.r.)	n.r.	[129]
	Liposomes with incorporated morin, targeted with mannose	DSPC:Chol (60:35)	127–137	[86]
	Liposomes with incorporated *p*-coumaric acid, targeted with mannose	DSPC:Chol (60:35)	114–124	[102]
	Liposomes with incorporated withaferin-A, targeted with mannose	DSPC:Chol (60:32.5)	150–155	[87]
	Liposomes with encapsulated or incorporated core peptide, targeted with RGD or HAP-1 peptides	DPPC:Chol:DSPE-PEG_2000_: DSPE-PEG_2000_-Mal (1.85:1.0:0.075:0.075)	95–105	[78]

^1^ Advanced to clinical trials (NIH identifier indicated in Section 5.1). n.r.—non-reported in the study.

**Table 2:** Drug delivery nanosystems currently in clinical trials for rheumatoid arthritis treatment.

Database	Drug Delivery Nanosystem	Identifier
USA National Library of Medicine	Polyethylene glycol (PEG)-liposomes containing prednisolone	NCT00241982 (phase II) and NCT02534896 (phase III)
	Recombinant adeno-associated virus vector	NCT00617032 (phase I); NCT00126724 (phase I/II); NCT02727764 (phase I) and NCT03445715 (phase I)
European Union Clinical Trials Register	PEG-liposomes containing prednisolone sodium phosphate (Nanocort^®^)	2015-002924-17 (phase III)

## Data Availability

No new data were created or analyzed in this study. Data sharing is not applicable to this article.

## Abbreviations

AIA—antigen-induced arthritis; ALP—alkaline phosphatase; ALT—alanine transferase; Apo2L/TRAIL—Apo2 ligand or tumor necrosis factor-related apoptosis-inducing ligand; AST—aspartate aminotransferase; CFA—complete Freund’s adjuvant-induced arthritis; CHEMS—cholesteryl hemisuccinate; Chol—cholesterol; CIA—collagen-induced arthritis; DCP—dicetylphosphate; DC_8,9_PC—1,2-bis(10,12-tricosadiynoyl)-sn-glycero-3-phosphocholine; Di-ART-GPC—dimeric artesunate-L-α-glycerophosphorylcholine conjugate; DMPC—1,2-dimyristoyl-sn-glycero-3-phosphocholine; DPPC—1,2-dipalmitoyl-sn-glycero-3- phosphocholine; DGS-NTA—1,2-dioleoyl-sn-glycero-3-{[N-(5-amino-1-carboxypentyl) -iminodiacetic acid]succinyl}; DMARDs—disease-modifying antirheumatic drugs; DMPA—1,2-dimyristoyl-sn-glycero-3-phosphate; DPPE—1,2-dipalmitoyl-sn-glycero- 3-phosphoethanolamine; DPPG — 1,2-dipalmitoyl-sn-glycero-3-(phospho-(1-rac-glycerol)); DOPC—1,2-dioleoyl-sn-glycero-3-phosphocholine; DOPE—1,2-dioleoyl-sn-glycero- 3-phosphoethanolamine; DSPC — 1,2-distearoyl-sn-glycero-3-phosphocholine; DSPG—1,2-distearoyl-sn-glycero-3-phospho-(1’-rac-glycerol); DSPE-PEG_2000_—1,2-distearoyl-sn- glycero-3-phosphoethanolamine-N-[methoxy(polyethylene glycol)-2000]; DSPE-PEG_2000_-CA—1,2-distearoyl-sn-glycero-3-phosphoethanolamine-N-[carboxy(polyethylene glycol)-2000]; DSPE-PEG_2000_-Mal—1,2-distearoyl-sn-glycero- 3-phosphoethanolamine-N-[maleimide(polyethylene glycol)-2000]; DSPE-PEG_2000_-NH_2_—1,2-distearoyl-sn-glycero-3-phosphoethanolamine-N-[amino(polyethylene glycol)-2000]; DSPE-PEG_3400_—1,2-distearoyl-sn-glycero- 3-phosphoethanolamine-N-[methoxy(polyethylene glycol)-3400]; e.c.—epicutaneous; E.E.—incorporation/encapsulation efficiency; EL—egg lecithin; EPC—egg phosphatidylcholine; FR-β—folate receptor beta; GMP—good manufacturing practice; GNRs—gold nanorods; HSPC—hydrogenated soybean phosphatidylcholine; H_2_O_2_—hydrogen peroxide; i.a.—intra-articular; IL—interleukin; i.m.—intramuscular; i.p.—intraperitoneal; i.v.—intravenous; JAK—Janus kinase; L.C.—loading capacity; LUVs—large unilamellar vesicles; MLVs—multilamellar vesicles; MMPs—metalloproteinases; MTX—methotrexate; nanoDDS—drug delivery nanosystems; NF-κB—nuclear factor-kappa B; ODNs—decoy oligodeoxynucleotides; PA—phosphatidic acid; PDI — polydispersity index; PEG—polyethylene glycol; PEG_400_-S—polyethylene glycol-400-stearate; PG—phosphatidylglycerol; PHEA-DODASuc—poly(L-hydroxyethyl asparagine)-N-succinyldioctadecylamine; POPC—1-palmitoyl-2-oleoyl-sn-glycero-3-phosphocholine; P-90G—Phospholipon® 90 G; NSAIDs—nonsteroidal anti-inflammatory drugs; O_2_—molecular oxygen; O_2_^•−^—superoxide radicals; RA—rheumatoid arthritis; RF—rheumatoid factor; RNS—reactive nitrogen species; ROS—reactive oxygen species; RPR209120—2-(3-[bis- (3-amino-propyl)-amino]-propylamino)-N-ditetradecylcarbamoylmethyl-acetamide; SA—stearylamine; s.c.—sub-cutaneous; SDS—sodium dodecyl sulfate; SL—soybean lecithin; SM—porcine brain sphingomyelin; SOD—superoxide dismutase; SPC—soybean phosphatidylcholine; SUVs—small unilamellar vesicles; S100—poly(ethyleneglycol) (100) monostearate; TLR—Toll-like receptor; TNF—tumor necrosis factor; TNF-α—tumor necrosis factor-alpha; VEGF—vascular endothelial growth factor; ζ—superficial charge.
